# Diagnostic value of IgE antibodies to ω-5 gliadin, HMW-glutenin and gliadins in wheat challenged children

**DOI:** 10.1186/2045-7022-3-S3-P116

**Published:** 2013-07-25

**Authors:** N Nilsson, S Sjolander, E Morita, M Berthold, G Hedlin, M Borres, C Nilsson

**Affiliations:** 1 Allergic and Pulmonary Diseases, Astrid Lindgrens Childrens Hospital, Stockholm, Sweden; 2 Thermo Fischer Scientific, Uppsala, Sweden; 3 Dep of Dermatology, Shimane University Faculty of Medicine, Izumo, Japan; 4 Dep of Clinical Science and Education, Sachs’ Children's Hospital, Stockholm, Sweden

## Background

Wheat is one of the six most common foods causing allergy in children. Tests used in clinical practice to diagnose wheat allergy have limited value in predicting allergic reactions when eating wheat. A poor correlation between the outcome of oral wheat-challenges and the levels of IgE-antibodies (IgE-ab) to wheat has been observed. The wheat allergen ω-5 gliadin has been found to facilitate the diagnosis of wheat dependent exercise induced anaphylaxis (WDEIA) in adults and also wheat allergy in children.

## Methods

Sixty children, with a doctor’s diagnose of wheat allergy, having IgE-ab to wheat and kept on a wheat elimination diet, were included in the study. All children went through an open oral wheat challenge. The initial dose was 0,05 g of bread followed by six steps of increasing doses every 30 minutes with a final dose of 17 g bread. Serum analysis of IgE-ab to wheat, ω -5 gliadin, recombinant HMW-glutenin and a native gliadin preparation containing α, β, γ and ω-5 gliadin using the ImmunoCAP technology was performed.

## Results

Thirty-one (52%) children reacted at the challenge: 16 developed asthma, 18 urticaria and/or atopic dermatitis, 9 rhinoconjunctivitis, 6 abdominal symptoms. IgE-ab to omega-5 gliadin showed a significant difference between the challenge positive and challenge negative children (p<0.0001). However, 10/31 children in the challenge positive group, had IgE-ab levels <0.35 kU/l to ω-5 gliadin. IgE-ab to HMW-glutenin and gliadin in children positive at challenge was detected in 30/31 and 29/31 children respectively. Also for HMW-glutenin and gliadin a statistically significant difference between challenge positive and challenge negative children was seen (p<0.0001).

## Conclusion

Our findings suggest that analysis of IgE-ab to wheat alone cannot predict the outcomes of oral wheat challenge but IgE-ab to ω-5 gliadin, HMW-glutenin and gliadin seem to be valuable markers for clinical wheat allergy in our study group.

## Disclosure of interest

None declared.

